# Influenza Polymerase Activity Correlates with the Strength of Interaction between Nucleoprotein and PB2 through the Host-Specific Residue K/E627

**DOI:** 10.1371/journal.pone.0036415

**Published:** 2012-05-03

**Authors:** Andy Ka-Leung Ng, Wai-Hon Chan, Sze-Ting Choi, Mandy Ka-Han Lam, Kwok-Fai Lau, Paul Kay-Sheung Chan, Shannon Wing-Ngor Au, Ervin Fodor, Pang-Chui Shaw

**Affiliations:** 1 Centre for Protein Science and Crystallography, School of Life Sciences, The Chinese University of Hong Kong, Shatin, Hong Kong SAR, China; 2 Department of Microbiology, Prince of Wales Hospital, The Chinese University of Hong Kong, Shatin, Hong Kong SAR, China; 3 Sir William Dunn School of Pathology, University of Oxford, Oxford, United Kingdom; University of Ottawa, Canada

## Abstract

The ribonucleoprotein (RNP) complex is the essential transcription-replication machinery of the influenza virus. It is composed of the trimeric polymerase (PA, PB1 and PB2), nucleoprotein (NP) and RNA. Elucidating the molecular mechanisms of RNP assembly is central to our understanding of the control of viral transcription and replication and the dependence of these processes on the host cell. In this report, we show, by RNP reconstitution assays and co-immunoprecipitation, that the interaction between NP and polymerase is crucial for the function of the RNP. The functional association of NP and polymerase involves the C-terminal ‘627’ domain of PB2 and it requires NP arginine-150 and either lysine-627 or arginine-630 of PB2. Using surface plasmon resonance, we demonstrate that the interaction between NP and PB2 takes place without the involvement of RNA. At 33, 37 and 41°C in mammalian cells, more positive charges at aa. 627 and 630 of PB2 lead to stronger NP-polymerase interaction, which directly correlates with the higher RNP activity. In conclusion, our study provides new information on the NP-PB2 interaction and shows that the strength of NP-polymerase interaction and the resulting RNP activity are promoted by the positive charges at aa. 627 and 630 of PB2.

## Introduction

Influenza is a contagious respiratory illness causing annual epidemics and occasional pandemics. Influenza epidemics cause between 250,000 and 500,000 deaths worldwide each year. Pandemics can be highly lethal; for instance, the Hong Kong Flu in 1968 killed approximately 1 million people worldwide [1]. The relatively mild and recently ended 2009 H1N1 pandemic resulted in over 18,449 deaths [2]. The 1997 influenza A H5N1 outbreak resulted in 6 deaths among 18 cases, with a high mortality rate of 33% [3]. Starting from 2003, the poultry-to-human transmission of this virus has been reported in many countries in Asia and Africa. There have been a total of 601 laboratory-confirmed human cases, of which 354 were fatal [4]. In view of influenza virus having the ability to cause epidemics and pandemics, a better understanding of how influenza virus functions at the molecular level is essential.

Influenza A virus is a negative-sense RNA virus. Its genome comprises eight segments of viral RNA (vRNA) encoding eleven or twelve proteins, including surface glycoproteins haemagglutinin (HA) and neuraminidase (NA), matrix protein M1, ion channel M2, non-structural proteins NS1, NS2 and the newly identified N40, nucleoprotein (NP), pro-apoptotic protein PB1-F2 and RNA-dependent RNA polymerase proteins PA, PB1 and PB2 (reviewed in ref. [5]). The ribonucleoprotein (RNP) complex is composed of the trimeric polymerase, NP and vRNA. The RNP complex is the essential element for transcription and replication of the viral genome [6]. During transcription, the cap-binding domain of PB2 first binds the 5′ 7-methylguanosine cap of the host pre-mRNA [7], [8]. The endonuclease domain of PA then cleaves it 9–15 nucleotides downstream of the cap [9], [10]. Then, PB1 uses this short piece of capped RNA as primer for initializing the transcription of mRNA [11], [12]. The vRNA is replicated via a complementary RNA (cRNA) intermediate in a primer-independent process. The role of NP is to provide a structural framework for vRNA and cRNA and it is also thought to be involved in regulating viral transcription and replication and act as an elongation factor (reviewed in ref. [13]).

RNPs are organized in a unique ‘7+1’ pattern in the virus [14] and appear as supercoiled structures under electron microscopy (EM) [15], [16]. The structure of a recombinant mini-RNP that contains a circular ring of nine NP molecules, a 248 nt-long RNA and the trimeric polymerase, has been studied extensively by single-particle imaging using EM. The first reconstruction reached a resolution of 27 Å at the NP ring and 36 Å at the polymerase [17]. The resolution of the polymerase was soon improved to 23 Å, with the domain positions of the polymerase subunits proposed [18]. Using cryo-EM, the resolutions at the NP ring and the polymerase were further raised to 12 and 18 Å respectively, which allowed the fitting of the recently determined crystal structures of NP and the partial PA-PB1 complex into the three-dimensional (3D) reconstruction [19]. The structure also suggested specific interactions of the PB1 and PB2 polymerase subunits with two molecules of NP adjacent to the polymerase. A number of advancements have been made in the x-ray and NMR structure determination of the different components of RNP since 2006. The atomic structures resolved now cover more than half of the sequence of the trimeric polymerase (reviewed in ref. [20]). In particular, we and others have determined the full protein structure of NP [21], [22]. NP was crystallized as a trimer and is organized into a head domain, a body domain and a tail loop. The structure of the PB2 ‘627’ domain, which contains the host-determining residue K/E627, has also been resolved [23], [24].

The phenotypic differences between human and avian influenza viruses have been extensively studied throughout the decades. Human influenza viruses replicate more efficiently in mammalian cells than avian cells, and the same is true for avian viruses in avian cells compared to mammalian cells [25]. This host range restriction is conferred in part by PB2 [26]–[28], in which seventeen host-determining residues have been identified [29]. The best characterized residue, at position 627, is predominantly a lysine in human influenza viruses and a glutamate in avian influenza viruses. Avian polymerase with E627 was shown to be selectively restricted in human cells [30], [31]. Avian viruses with an E627K mutation have shown improved growth in human cells and enhanced virulence in mice. PB2 with K627 was also shown to replicate more efficiently at 33°C (i.e. upper respiratory tract in human) than PB2 with E627 [32]–[34].

Before the EM 3D reconstruction of the RNP suggesting specific polymerase-NP interaction, there was already biochemical evidence for such interaction. NP was found to interact with PB1 and PB2 but not PA, in both a recombinant system and in virus-infected cells [35], [36]. Three regions of NP (aa. 1–161, 255–340 and 340–465) were found to interact independently with PB2, while the C-terminus of NP (aa. 465–498) was found to inhibit NP-PB2 binding [35]. Later, two PB2 fragments (N-terminal aa. 1–269 and C-terminal aa. 580–683) were also identified as being responsible for NP-binding [37]. There are, however, considerable overlaps of the NP- and PB1- binding sites on PB2. Two recent functional studies have suggested that the E627K mutation can strengthen the NP-polymerase interaction in avian influenza virus RNPs in human cells [38], [39]. In the present report, we identify residues in NP and PB2 that mediate NP-polymerase interaction and show how the strength of the interaction is related to the RNP activity.

## Results

### A single point mutation R150A in NP abolishes RNP activity in an H5 polymerase background, but not an H1 background

**Figure 1:**
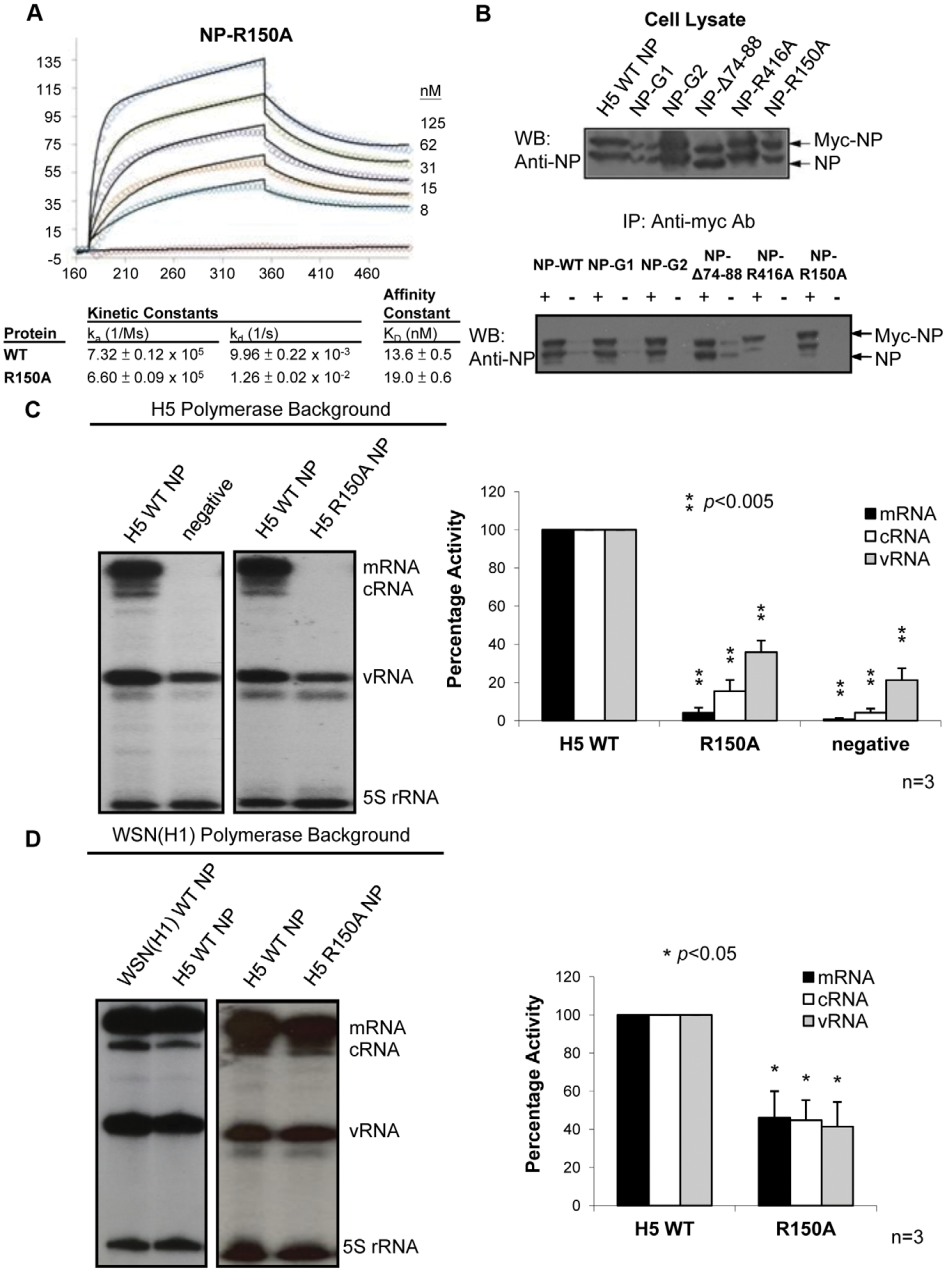
The R150A NP mutant shows different activities in H5 and WSN(H1) polymerase backgrounds. (A) SPR of different concentrations of NP R150A mutant against immobilized RNA. (B) Co-immunoprecipitation of flag-tagged NP mutants with their myc-tagged counterparts. ‘+’ refers to the presence of the anti-myc antibodies while ‘−’ indicates their absence. (C) The wild-type and R150A mutant NP were subjected to RNP reconstitution assay in an H5 background and viral RNA (NA) levels were quantified by primer extension. A representative result of three independent experiments is shown. RNA levels of the NP R150A mutant were compared to those of wild-type NP, which was set to 100%. 5S rRNA was used to normalize the m-, c- and v-RNA levels. The quantitation represents the mean percentage ± standard deviations from three experiments. (D) RNP reconstitution assay of wild-type and R150A mutant NP in an WSN(H1) background (*, *P*<0.05; **, *P*<0.005).

The resolution of the NP crystal structure [21], [22] has enabled the dissection of its functional domains. We have previously identified, by surface plasmon resonance (SPR), three regions in NP (G1 [R74, R75, R174, R175, R221], G2 [R150, R152, R156, R162] and a flexible basic loop [aa. 74–88]) which are involved in RNA binding [21]. During the investigation, we have identified an NP mutant (NP-R150A) which had normal RNA binding affinity (Fig. 1A) and NP-NP interaction (Fig. 1B), but displayed different RNP activities when analysed with polymerases from different influenza virus subtypes.

The R150A H5 NP mutant plasmid was co-transfected with plasmids expressing polymerase proteins [either from A/HongKong/156/97(H5N1) or A/WSN/33(H1N1)] and a reporter plasmid expressing NA vRNA into 293T cells. The amounts of NP plasmids were adjusted to give similar protein expression levels. A plasmid expressing wild-type H5 NP was used as the positive control, while an empty plasmid was the negative control. At 48 h post-transfection, total RNA was extracted, and the vRNA, cRNA and mRNA levels of the reporter gene were quantified by a primer extension assay, followed by polyacrylamide gel electrophoresis and autoradiography (Fig. 1C and 1D). The various RNA levels were normalized to the internal 5 S rRNA control and compared with those of the wild-type NP.

In an H5 polymerase background, the R150A mutation in NP has led to a total loss in its RNP activity (Fig. 1C). In an H1 polymerase background, the R150A mutation could however retain about half of its activity compared to wild-type (Fig. 1D). This suggests that the defective phenotype of the NP R150A mutation is strain-specific.

### NP R150A associates with the polymerase complex and forms an active RNP in the presence of WSN(H1) PB2, but not H5 PB2

**Figure 2:**
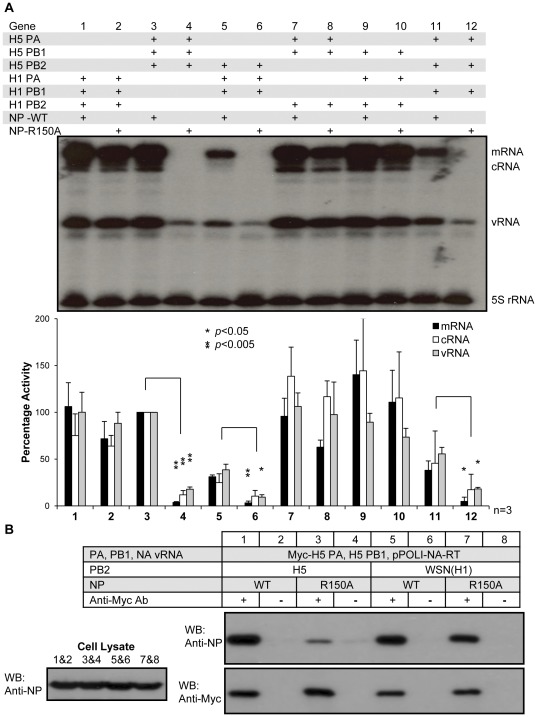
The R150A NP mutant forms a functional association with polymerases containing WSN(H1) PB2. (A) RNP reconstitution assay with gene-swapped polymerases and wild-type or R150A mutant NP. RNA levels were compared to those with wild-type NP in H5 polymerase background, which was set to 100% (Lane 3). A representative result of three independent experiments is shown. P-values are calculated versus wild-type NP for each set of polymerase combination (*, *P*<0.05; **, *P*<0.005). (B) Co-immunoprecipitation of wild-type and R150A mutant NP with Myc-tagged polymerase carrying either H5 or WSN(H1) PB2.

Next, we investigated why the defective NP R150A phenotype was strain-specific. We swapped the polymerase subunits between H5 and WSN(H1) and performed RNP reconstitution assays with either wild-type NP or the R150A mutant (Fig. 2A). All combinations of polymerases with wild-type NP gave detectable RNP activity, although some were less active than others (Fig. 2A, odd numbered lanes). The variation of RNP activities may be due to differences in the compatibility of the polymerase subunits from H1 and H5 subtypes. On the other hand, in the presence of NP R150A, active RNP activity was obtained when PB2 was from WSN(H1) (lanes 2, 8, 10) but not from the H5 subtype (lanes 4, 6, 12). PB2 is therefore the determining factor responsible for the differential phenotypes of the NP R150A mutant in WSN(H1) and H5 polymerase backgrounds.

Why does the NP R150A form an active RNP with WSN(H1) PB2 but not H5 PB2? We hypothesized that this may be related to the interaction between NP and the polymerase complex. To test this, NP wild-type or the NP R150A mutant were co-expressed with the NA vRNA, H5 PB1, WSN(H1) or H5 PB2 and myc-tagged H5 PA in 293T cells. Co-immunoprecipitation was performed at 48 h post-transfection with anti-myc antibody, followed by detection of NP and the myc-tagged PA by western blotting (Fig. 2B). Wild-type NP was co-immunoprecipitated in the presence of myc-tagged polymerase complex containing either H5 or WSN(H1) PB2. However, mutation of NP R150A significantly reduced its interaction with the polymerase complex containing H5 PB2, but not WSN(H1) PB2. Similar results were obtained in the absence of NA vRNA (Fig. S1).

### Replacing the C-terminus of H5 PB2 with that of WSN(H1) PB2 restores the RNP activity and NP-polymerase interaction of the NP R150A mutant

**Figure 3:**
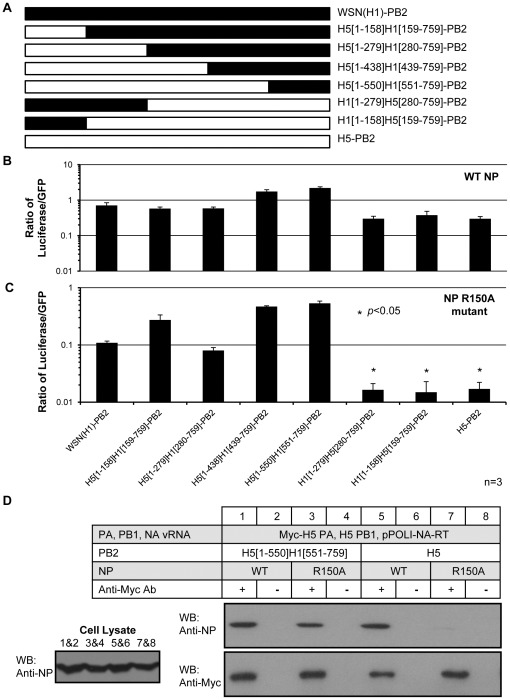
Polymerase activity assay and co-immunoprecipitation of domain-swapped PB2 mutants. (A) Construct design of domain-swapped PB2 mutants. (B and C) RNP reconstitution assay of domain-swapped PB2 with (B) wild-type and (C) R150A mutant NP. A plasmid encoding GFP was co-transfected for data normalization purposes. The RNP activity was reported as the ratio of luciferase activity to the GFP level. The RNP activities of the domain-swapped PB2 proteins were compared to that of WSN(H1)-PB2. The bar chart represents the mean ratio ± standard deviations from three independent experiments (*, *P*<0.05). (D) Co-immunoprecipitation of wild-type and R150A mutant NP with Myc-tagged polymerase carrying either H5 or H5[1–550]H1[551–759] PB2.

Alignment of the WSN(H1) and H5 PB2 amino acid sequences revealed an identity of 94% (results not shown). We set out to investigate which region in the WSN(H1) PB2 was responsible for the interaction with NP by creating a series of chimeric WSN(H1) and H5 PB2 proteins (Fig. 3A). A luciferase reporter assay [40] was used to measure the overall RNP activity. A plasmid encoding a vRNA-like luciferase gene was co-transfected into 293T cells with either wild-type or R150A NP, PA, PB1 and the various PB2 constructs. A plasmid encoding green fluorescent protein (GFP) was also co-transfected as a control. The RNP activity was reported as the ratio of luciferase activity to GFP level. Transfection of all six chimeric PB2 mutants with wild-type NP resulted in detectable RNP activity, indicating that they were functional (Fig. 3B). As observed before, RNP with wild-type H5 PB2 and NP R150A mutation did not give significant activity (Fig. 3C, H5-PB2). Replacing the N-terminal region of H5 PB2 with aa. 1–279 of WSN(H1) PB2 did not increase the RNP activity (Fig. 3C, H1[1–279]H5[280–759]-PB2). This indicates that the N-terminus of WSN(H1) PB2 could not rescue the defective phenotype of the NP R150A. On the other hand, replacing aa. 551–759 of H5 PB2 by the corresponding C-terminal region of WSN(H1) PB2 rendered the RNP active (Fig. 3C, H5[1–550]H1[551–759]-PB2).

Co-immunoprecipitation of wild-type or R150A NP with the H5[1–550]H1[551–759]-PB2 chimeric protein was then performed. It was found that H5[1–550]H1[551–759]-PB2 could restore the NP-polymerase interaction with the NP R150A mutant (Fig. 3D). Taken together, these experiments showed that the C-terminus of WSN(H1) PB2 can overcome the inhibitory effect of NP R150A mutation in the background of an H5 polymerase.

### Residues 627 and 630 in PB2 are crucial for the NP-polymerase interaction in mammalian cells

**Figure 4:**
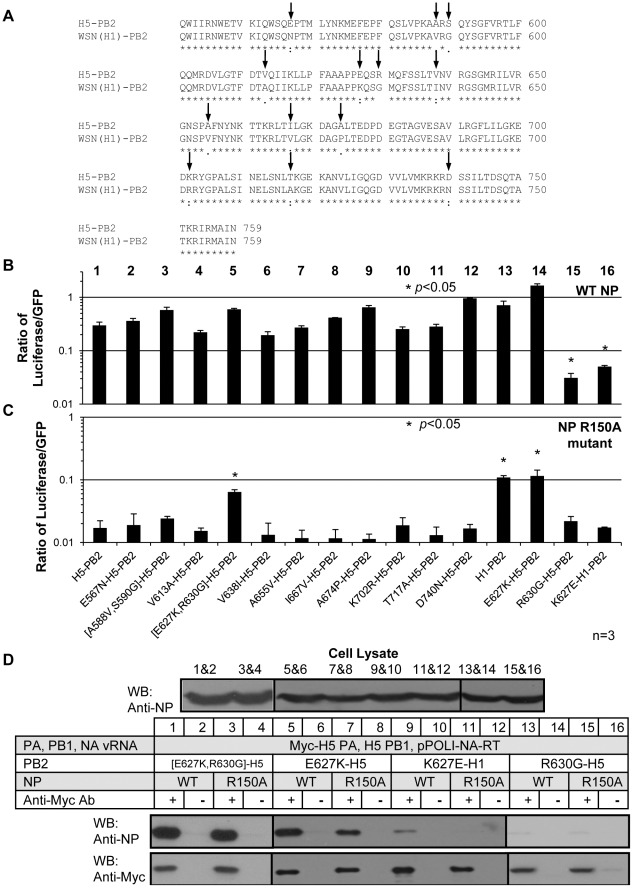
K627 and R630 are crucial for the RNP activity and NP-PB2 interaction. (A) Sequence alignment of H5 and WSN(H1) PB2 C-terminal region (aa. 551–759). The arrows denote the differences. (B and C) RNP reconstitution assay of PB2 point mutants with (B) wild-type or (C) R150A mutant NP. The mean RNP activities from three independent experiments of the PB2 mutants were compared to those of wild-type H5-PB2 (*, *P*<0.05). (D) Co-immunoprecipitation of wild-type and R150A mutant NP with Myc-tagged polymerase variants in 293T cells at 37°C.

Sequence alignment of the C-terminus of PB2 of the two strains was performed to identify the particular residues in the C-terminus of PB2 (aa. 551–759) which caused the differential phenotypes in the presence of the NP R150A mutant. 13 polymorphic amino acid residues were found between the two strains (Fig. 4A). We constructed nine single-point and two double-point H5 PB2 mutants with polymorphic amino acid residues from WSN(H1) PB2. The expression levels of these PB2 mutants were normalized and the RNP activities of the mutants were measured using a luciferase reporter assay with wild-type NP (Fig. 4B, bars 1–12) or NP R150A (Fig. 4C, bars 1–12).

All eleven H5 PB2 mutants could form active RNPs with wild-type NP (Fig. 4B, bars 1–12). However, only one double-point mutant ([E627K,R630G]-H5-PB2) could recover the RNP activity in the presence of the NP R150A mutant (Fig. 4C, [E627K,R630G]-H5-PB2). To test whether K627 or G630 of WSN(H1) PB2 was the key residue, we constructed two additional point mutants (E627K-H5-PB2 and R630G-H5-PB2). It was found that the E627K mutation in H5 PB2 gave normal RNP activity with wild-type NP and could compensate for the NP R150A mutation (Fig. 4B and 4C, bar 14). A co-immunoprecipitation experiment also demonstrated that the E627K-H5-PB2 restored the NP-polymerase interaction of NP R150A mutant (Fig. 4D, lanes 5–8).

Surprisingly, the other mutant R630G-H5-PB2, which contains E627, could not produce significantly active RNP even with wild-type NP (Fig. 4B, bar 15), although the mutant was expressed to similar level as the wild-type PB2 in 293T cells (data not shown). We also changed K627 to E in WSN(H1) PB2, which has already got G630 (K627E-H1-PB2). RNP activity with wild-type NP was found to be greatly reduced (Fig. 4B, bar 16). Co-immunoprecipitation experiments also showed that both mutants (R630G-H5-PB2 and K627E-H1-PB2) lost nearly all NP-polymerase interaction with wild-type NP (Fig. 4D, lanes 9–16).

From the above observations (summarized in Table 1), we can make the following conclusion. Residues at positions 627 and 630, but not the origin of PB2, determine the NP-polymerase affinity and RNP activity (rows 1–8). With the WSN(H1) PB2 genotype (K627 and G630), both wild-type NP and R150A NP could form a functional RNP with similar polymerase activity (rows 1–4). With the H5 PB2 genotype (E627 and R630), only the wild-type NP but not the R150A NP could form a functional RNP (rows 9–10). This showed that a positively charged residue, either at position 627 or 630, is required to form a functional RNP. Without a positively charged amino acid residue at either of these positions, PB2 is inactive in producing functional RNP and NP-polymerase interactions (rows 5–8). In addition, it is crucial to have an arginine residue at position 150 in NP if there is a positively charged residue at position 630, but not at position 627 of PB2 (rows 1–2, 9–10). This suggests that polymerase with K627-containing PB2 does not require R150 in NP for the interaction with NP.

**Table 1:** Summary of the key findings.

	PB2			NP		Phenotype		
No.	Variants	aa. 627	aa. 630	Variants	aa. 150	Polymerase activity (Luciferase Assay)	NP-polymerase interaction (Co-IP)	NP-PB2 ‘627-domain’ interaction (SPR)
1	[E627K, R630G] -H5 PB2	K	G	WT	R	Active	Yes	Yes
2				R150A	A	Active	Yes	Yes
3	WT-H1 PB2	K	G	WT	R	Active	Yes	n/a
4				R150A	A	Active	Yes	n/a
5	R630G-H5 PB2	E	G	WT	R	Inactive	No	No
6				R150A	A	Inactive	No	No
7	K627E-H1 PB2	E	G	WT	R	Inactive	Very weak	n/a
8				R150A	A	Inactive	No	n/a
9	WT-H5 PB2	E	R	WT	R	Active	Yes	Yes
10				R150A	A	Inactive	Very weak	Largely weakened

Note:

‘n/a’ refers to not available.

**Figure 5:**
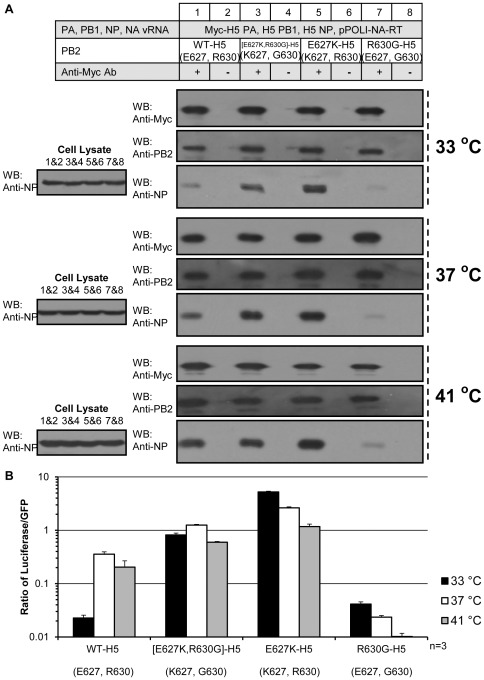
Temperature-dependent NP-PB2 interaction and polymerase activity. (A) Co-immunoprecipitation of wild-type NP by Myc-tagged polymerase carrying different PB2 at 33°C, 37°C and 41°C. (B) RNP reconstitution assay of PB2 point mutants at 33°C, 37°C and 41°C.

To find the interaction of polymerase with NP and the RNP activities at different temperatures, co-immunoprecipitation of NP and luciferase assays on PB2 with E627/R630 (wild-type H5), E627/G630, K627/R630, K627/G630 were performed at 33, 37 and 41°C in mammalian cells (Fig. 5). Similar levels of the different PB2 variants were co-immunoprecipitated by myc-tagged PA at all tested temperatures (Fig. 5A). In the absence of positive charges at positions 627 and 630, the affinity to NP and the RNP activity were found to be low at all tested temperatures (Fig. 5A, lane 7; Fig. 5B, R630G-H5). On the other hand, polymerase carrying wild-type H5 PB2, which has E627 and R630, showed low affinity to NP and low RNP activity at 33°C, but increased significantly at 37 and 41°C (Fig. 5A, lane 1; Fig. 5B, WT-H5). Both the wild-type H5 PB2 and [E627K,R630G]-H5 PB2 carry one positive charge at the concerned region, the former with R630 while the latter with K627. K627 led to more efficient interaction with NP and higher RNP activity than R630 (Fig. 5A, lanes 1 and 3; Fig. 5B, WT-H5 and [E627K,R630G]-H5). Compared to the polymerase carrying wild-type H5 PB2, more positive charges at aa. 627 and 630 gave a higher affinity with NP and a higher RNP activity at all tested temperatures (Fig. 5A, lanes 1 and 5; Fig. 5B, WT-H5 and E627K-H5).

### The ‘627-domain’ of PB2 directly interacts with NP, without the involvement of RNA

**Figure 6:**
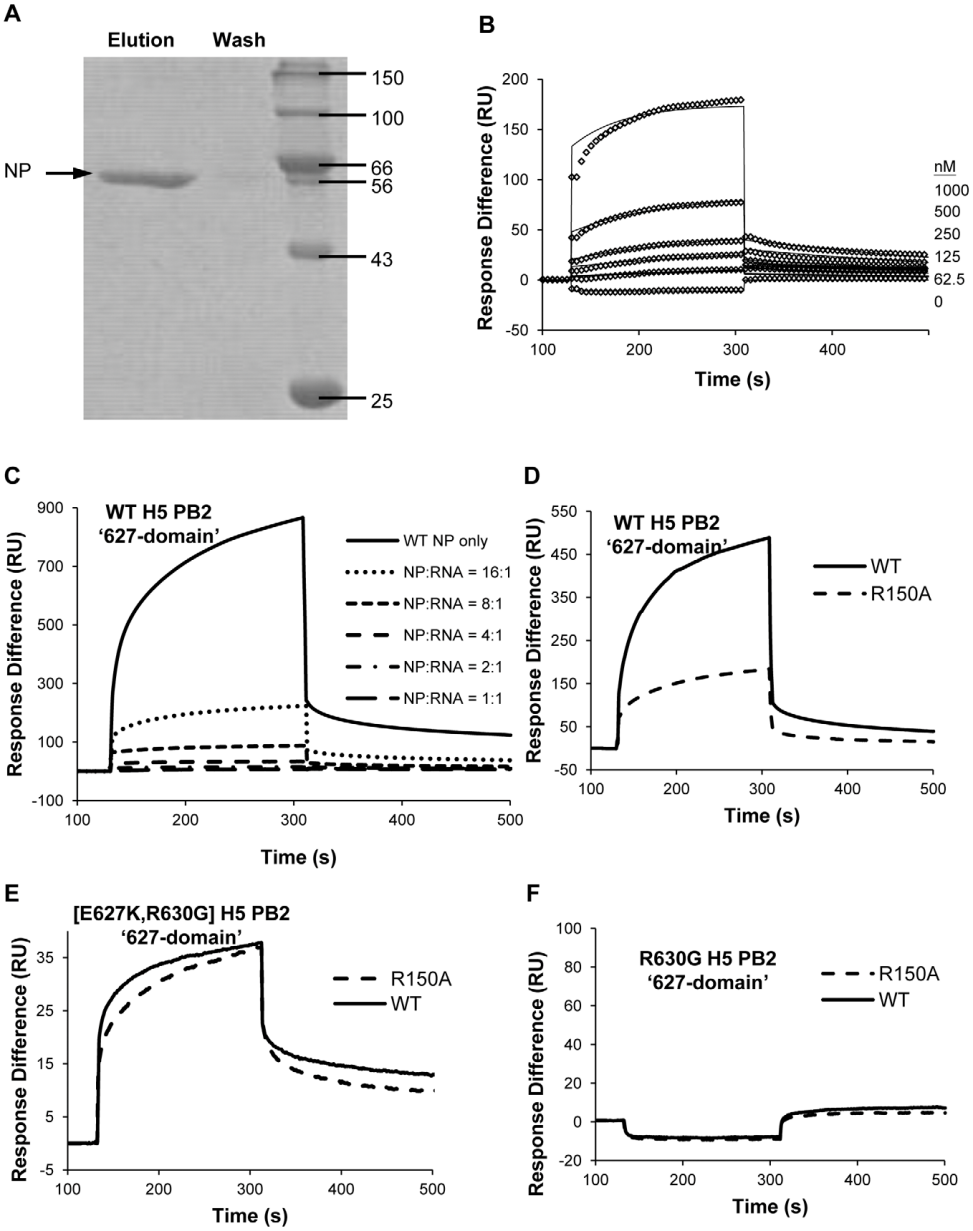
Biophysical characterization of the NP-PB2 ‘627-domain’ interactions. (A) Pull down assay to analyze NP-PB2 interaction. Purified PB2 ‘627-domain’ was covalently immobilized onto an NHS column. Purified NP was then applied and eluted after extensive washing. (B) SPR analysis of NP-PB2 interaction. Wild-type PB2 ’627-domain’ was immobilized on a CM5 chip. NP in increased concentration was applied to the chip surface. The response differences of experimental and control flow cells are reported (individual diamonds). Solid lines are the fitted curves. (C) Wild-type NP alone (2 µM), and NP with different molar ratios of RNA were passed through the chip. (D, E and F) Wild-type and R150A mutant NP were analyzed with immobilized (D) WT, (E) [E627K,R630G] and (F) R630G H5 PB2 ‘627-domain’ by SPR.

After the identification of the crucial residues in PB2 (K627 and R630) and NP (R150) required for NP-polymerase interaction, we then investigated how NP and PB2 interact. The NP-PB2 interaction could be either direct or indirect, or even involving RNA, as both NP and PB2 have been shown to possess RNA binding activities [21], [24]. To understand the mode of interaction, we employed *in vitro* pull-down assays and SPR with BIAcore 3000. We expressed NP and the PB2 ‘627-domain’ (aa. 538–693) in *E. coli* and purified the protein to high homogeneity using established protocols [21], [23]. The purified PB2 ‘627-domain’ was covalently immobilized on an NHS-column. The column was then incubated with purified NP. After extensive washing, the bound protein, if any, was eluted with a high salt buffer. It was found that the PB2 ‘627-domain’ interacts with NP (Fig. 6A). An empty column was also incubated with purified NP as a negative control, with no NP being eluted (data not shown).

The purified PB2 ‘627-domain’ was also immobilized on a CM5 chip by amine coupling. Purified NP was allowed to pass through the chip in a concentration series (Fig. 6B). The kinetic parameters were measured, the binding curves were fitted and the affinity of the interaction was calculated. The best-fitted model is the ‘1∶1 Langmuir model", which is consistent with the phenomenon that one RNP contains one copy of PB2. Wild-type NP bound wild-type H5 PB2 ‘627-domain’ through direct protein-protein interaction, with an affinity of 252 nM (Table 2). To determine if RNA played a role in the interaction, a 24-nt 2′-*O*-methylated RNA, which confers RNase resistance, was mixed with NP in different molar ratios. The NP/RNA mixture was allowed to pass through the chip and it was found that the NP-PB2 ‘627-domain’ interaction decreased with increasing amount of RNA (Fig. 6C). Thus, the presence of RNA caused an inhibitory effect suggesting that RNA is not involved in the interaction.

**Table 2:** Kinetics and affinity constants of NP-PB2 interaction.

H5 PB2	NP	Association Rate k_a_ (M^−1^ s^−1^)^2^	Dissociation Rate k_d_ (s^−1^)^2^	Affinity K_D_ (nM)^1^^,^ 2	Fold change^3^
WT	WT	1.34±0.02×10^4^	3.37±0.08×10^−3^	252±8	−
WT	R150A	3.26±0.12×10^3^	1.15±0.03×10^−2^	3510±39	−12.9
[E627K,R630G]	WT	1.05±0.01×10^4^	1.76±0.06×10^−3^	168±6	+0.5
[E627K,R630G]	R150A	6.02±0.09×10^3^	3.56±0.08×10^−3^	591±16	−1.3

Note:

1 K_D_ = k_d_/k_a_.

2 The errors of k_a_ and k_d_ represent the standard error while the error of K_D_ represents the maximum error from each experiment.

3 Fold change is given with respect to the WT, ‘+’ represents fold increase while ‘−’ represents fold decrease.

To test if the crucial residues (PB2 K627, R630 and NP R150) for NP-polymerase binding and RNP activities are related to this direct NP-PB2 ‘627-domain’ interaction, we cloned the [E627K,R630G] and R630G mutations separately into H5 PB2 ‘627-domain’ for bacterial protein expression and purification. The [E627K,R630G] mutant mimics the genotype of WSN(H1) PB2. The binding affinities of these H5 PB2 ‘627-domain’ mutants with NP were analyzed (Table 2). It was found that the NP R150A variant bound 12.9 fold weaker than wild-type NP to the wild-type H5 PB2 ‘627-domain’ (Fig. 6D, Table 2). This correlates with the defective phenotype of NP R150A mutant with H5 PB2 (Fig. 2A and 2B). When the H5 PB2 ‘627-domain’ [E627K,R630G] variant was immobilized on the sensor chip, both wild-type and mutant R150A NP showed high binding affinities (Fig. 6E, Table 2). This again correlates with the RNP activity and NP-polymerase interaction of NP R150A mutant with [E627K,R630G]-H5-PB2 (Fig. 4C and 4D). On the other hand, neither wild-type nor the R150A NP mutant interacted with immobilized H5 PB2 R630G (Fig. 6F). This also agrees with the lack of RNP activity and NP-polymerase interaction of R630G-H5-PB2 (Fig. 4B, 4C and 4D). Therefore, we conclude that the direct protein-protein interaction of NP and PB2 ‘627-domain’ plays a crucial role in the NP-polymerase interaction as well as the activity of the RNP complex.

## Discussion

In this report, the amino acid residues K627 and R630 in the ’627-domain’ of PB2 were found to be important for mediating the interaction between NP and polymerase. In addition, a region of NP involving the conserved amino acid residue 150 was also found to be important in mediating such interaction. These results facilitate our understanding of the molecular mechanisms of RNP assembly of influenza A viruses.

Using H5 and WSN strains as models, our data indicate that either K627 or R630 in PB2 can mediate a functional NP-polymerase interaction. Results from virus replication assays also support this conclusion. Avian influenza virus isolates HK156 and MZ237, which contain E627 and R630, were shown to form plaques and possess transcription-replication activities at 37°C in 293T cells [38]. A reassortant WSN virus containing polymerase and NP genes from the avian isolate MZ reached similar viral titers as a reassortant WSN virus with polymerase and NP genes from the human isolate P908 at 48 hpi on MDCK cells, although it exhibited slower growth during the first 30 hpi [39]. These studies showed that viruses containing R630, but not K627, in PB2 could replicate efficiently in mammalian cells. The WSN virus with PB2 containing K627 and G630, was also found to produce high virus titer at 37°C in MDCK cells. This virus also had high polymerase activity in 293T and Cos7 cells [30]. However, when both K627 and R630 were absent, the virus could hardly replicate in mammalian cells. The WSN virus with PB2 K627E mutation and G630 gave low polymerase activity and formed very few plaques in MDCK cells [30]. Interestingly, when PB2 with E627 and G630 was co-expressed with NP, an interaction was observed [30]. However, it was shown previously that the N-terminal 1–269 amino acids of PB2 can also interact with NP [37] and we speculate that the observed interaction could have been mediated primarily by the N-terminal region of PB2. When the whole RNP complex is expressed, the interaction between the polymerase carrying PB2 K627E mutation and NP in mammalian cells is weak, as observed by us (Fig. 4B) and other [30].

The PB2 ‘627-domain’ construct used in the SPR experiment lacks the N-terminal region of PB2, which was shown to bind NP [37]. Yet the PB2 ‘627-domain’ is independently folded and residue 627 at the domain was shown to affect the NP-polymerase interaction [38]. According to the atomic structure of the PB2 ‘627-domain’ [23], [24], aa. 627 and 630 are on a flexible loop, and the conformation is likely to change upon interaction with NP. How exactly these residues contribute to the interaction would need the elucidation of the structure of the PB2-NP complex. Concerning the involvement of RNA, we showed by SPR experiments that RNA in fact inhibits the NP-PB2 ‘627-domain’ interaction, suggesting that RNA is not involved in the interaction between PB2 and NP in RNP.

There are more than 10,000 PB2 sequences from various influenza strains in the NCBI database. Avian viruses and the 2009 H1N1 pandemic strain have the combination of E627 and R630 (61.0% of all PB2 sequences). Human viruses typically have the combination of K627 and R630 (38.2% of all PB2 sequences), with the exception of the 2009 H1N1 pandemic. It is noted that the combination of K627 and G630 is confined to the WSN strain.

Avian influenza viruses carrying PB2 E627 do not replicate efficiently at 33°C in mammalian cells [32]–[34]. We have found that polymerase from human H5N1 isolate with PB2 E627 has lower RNP activity at 33°C than those with PB2 mutated to K627 (Fig. 5). With PB2 E627K mutation, the avian viruses have improved growth in mammalian cells and enhanced virulence in mice and possibly humans [32]–[34]. The phenotypic differences of K/E627 are likely brought by either an inhibitory co-factor on E627 or an adaptive co-factor on K627 in human cells [30], [31].

We observe at all tested temperatures that stronger the interaction between NP and polymerase, better their RNP activities (Fig. 5). At temperatures in both the mammalian respiratory tract (33°C) and the avian intestinal tract (41°C), polymerase with more positive charges at PB2 aa. 627 and 630 interacts better with NP and confers higher RNP activity (Fig. 5). This coincides with the previous findings that (1) more NP was co-purified by strep-PB2 tagged human-like polymerase than avian-like polymerase [39]; and (2) E627K mutation in an avian PB2 strengthened the NP-polymerase interaction in human cells [38].

To conclude, this work contributes to our understanding of how NP interacts with PB2 and polymerase in influenza virus RNP. A region of NP involving amino acid residue 150 was found to interact with K627 or R630 of PB2. The direct correlation of the strength of NP-polymerase interaction and the RNP activity at different physiological temperatures was established. These provide new information of the requirements of the replication machinery of the influenza viruses.

## Materials and Methods

### Biological Materials

The 293T cell line (ATCC, Manassas, VA, USA) was cultivated in minimal essential medium (MEM) (Invitrogen, Carlsbad, CA, USA) with 10% fetal calf serum (Invitrogen). Anti-NP and anti-PB2 sera were prepared by immunizing rabbits with purified NP and the N-terminus of PB2. Anti-myc antibody (Cell Signaling, Danvers, MA, USA), anti-beta-actin antibody (GenScript, Piscataway, NJ, USA) were purchased commercially. Plasmids pcDNA-PB1, pcDNA-PB2, pcDNA-PA, pcDNA-NP and pPOLI-NA-RT have been described previously [41], [42]. pEGFP and pPolI-Luc-RT are gifts from L.L.M. Poon and have been described [40]. NP and PB2 mutants were cloned into pcDNA3 (Invitrogen) for the expression of untagged NP and PB2 variants in 293T cells. H5 PA was cloned into pcDNA3.1/myc-His (Invitrogen) for the expression of myc-tagged PA in 293T cells.

### RNA analysis by primer-extension assay

Human kidney 293T cells were used to reconstitute RNP complexes. 1 µg of each of the pcDNA-PB1, pcDNA-PB2, pcDNA-PA, pcDNA-NP, and pPOLI-NA-RT plasmids were diluted to a total volume of 250 µl in OptiMEM (Invitrogen) and subsequently added to a mix of 7.5 µl of Lipofectamine 2000 (Invitrogen) in 250 µl OptiMEM. The transfection mixture was incubated for 30 minutes before adding to 1.5 ml (about 10^6^ cells) 293T cells in suspension in minimal essential medium (MEM) containing 10% fetal calf serum in 35-mm dishes. Cells were incubated at 37°C, harvested 48 hours post-transfection, and total RNA was extracted by TRIzol reagent (Invitrogen).

Primer extension assays were performed as described previously [42], [43]. Briefly, an excess of DNA primer (about 10^5^ cpm), labeled at its 5′ end with ^32^P, was mixed with 5 µg of total RNA in 5 µl of water, and denatured at 95°C for 3 minutes. The mixture was cooled on ice and subsequently incubated at 45°C for 1 hour with the addition of 50 U SuperScript II RNase H^−^ reverse transcriptase (Invitrogen) in First Strand Buffer (Invitrogen). Two NA gene-specific primers and one 5 S ribosomal RNA primer (used as an internal control) were used in the same reverse transcription reaction: 5′-GGACTAGTGGGAGCATCAT-3′ (to detect vRNA), 5′-TCCAGTATGGTTTTGATTTCCG-3′ (to detect mRNA and cRNA) and 5′-TCCCAGGCGGTCTCCCATCC-3′ (to detect 5 S rRNA). Reactions were stopped by the addition of 8 µl 90% formamide and heating at 95°C for 3 minutes. Transcription products were analyzed on 6% polyacrylamide gels containing 7 M urea in TBE buffer and detected by autoradiography. Phosphorimage analysis by ImageQuant TL (GE Healthcare, Waukesha, WI, USA) was used for quantification. An unpaired Student's *t*-test was used for analysis of significance.

### Polymerase activity analysis by luciferase assay

0.125 µg of each of the pcDNA-PB1, pcDNA-PB2, pcDNA-PA, pcDNA-NP, and pPOLI-Luc-RT plasmids and 0.0625 µg of pEGFP plasmid were diluted to a total volume of 12.5 µl in OptiMEM (Invitrogen) and subsequently added to a mix of 1.05 µl of Lipofectamine 2000 (Invitrogen) in 12.5 µl OptiMEM. The transfection mixture was incubated for 30 minutes in the 96-well plate before 75 µl (about 10^5^ cells) 293T cells in minimal essential medium (MEM) containing 10% fetal calf serum was added into the well. GFP fluorescent signal was first measured 48 h post-transfection by Victor 2 Multilabel plate reader (Perkin Elmer, Waltham, MA, USA). Afterwards, cells were lysed by Steady-Glo assay reagent (Promega, Madison, WI, USA) for 5 minutes, and the luminescence signal was measured. The polymerase activity was reported as the ratio of GFP signal to luminescence signal. An unpaired Student's *t*-test was used for analysis of significance.

### Co-immunoprecipitation of NP-polymerase and NP-NP

1 ug each of Myc-tagged PA, untagged PB1, PB2 and NP and pPol-NA-RT plasmids were transfected into 10^6^ human kidney 293T cells in suspension. Co-immunoprecipitation was performed 48-hours post-transfection. Cells were resuspended in 50 mM Tris-HCl (pH 7.6), 150 mM NaCl, 1 mM EDTA, 1% Triton X-100 (co-IP buffer) and lysed by sonication. The lysate was centrifuged at 16000 g for 10 minutes at 4°C. The supernatant was incubated at 4°C overnight with or without anti-myc antibody. The mixture was then incubated with protein-A beads for 1.5 hours at 4°C with shaking. The beads were centrifuged and washed with co-IP buffer four times before being boiled in SDS-loading dye and analyzed by western blotting. Experiment to study NP homo-oligomerization was performed as described [44]. 1 ug each of untagged and myc-tagged NP plasmids were transfected into the 293T cells. 150 U RNaseA (Sigma) was added to the supernatant after centrifugation.

### Expression and purification of NP

Maltose binding protein (MBP)-tagged NP was expressed in *Escherichia coli* BL21(DE3)pLysS. The cells were lysed by sonication, and the lysate was passed through an amylose column (New England Biolabs, Ipswich, MA, USA). Bound protein was eluted with a 0–20 mM maltose gradient in 20 mM sodium phosphate (pH 6.5) and 150 mM NaCl. The eluate was incubated with thrombin (100 U) (Sigma) and RNase A (100 U) (Sigma) at 4°C overnight to remove MBP from NP and then passed through a heparin HP column (GE Healthcare). NP was eluted with a 0–1.5 M NaCl gradient in the same buffer. Gel filtration was performed with Superdex 200 (GE Healthcare). RNase A was removed after passing through heparin HP column and gel filtration. NP R150A mutant was generated by site-directed mutagenesis of the wild-type pRSETMBP-NP plasmid [21] following a standard protocol, and was purified as described for the wild-type protein.

### Expression and purification of the PB2 ‘627-domain’

The method has been described previously [23]. Briefly, His-tagged PB2 ‘627-domain’ was expressed in *Escherichia coli* BL21(DE3). The cells were lysed by sonication in lysis buffer (30 mM Tris-HCl, 200 mM NaCl, pH 7.0). The lysate was then passed through a His column (GE Healthcare). Bound protein was washed with lysis buffer, followed by sequential washes with 1 M NaCl, 50 mM imidazole and 75 mM imidazole in lysis buffer to reduce non-specific binding. His-tagged PB2 ‘627-domain’ was eluted with 500 mM imidazole in lysis buffer. Gel filtration was then performed with Superdex 75 (GE Healthcare) in lysis buffer or in phosphate buffered saline (PBS).

### In vitro pull down assay

Purified wild-type PB2 ‘627-domain’ was covalently immobilized on an NHS column, according to the manufacturer's instructions (GE Healthcare). Wild-type NP in PBS was then applied to the column and incubated for 1 h at room temperature. The column was washed with at least 20 column volumes of PBS. Bound protein was eluted with 1.5 M NaCl in PBS and analyzed by SDS-PAGE.

### SPR analysis of NP-PB2 ‘627-domain’ and NP-RNA interaction

Purified wild-type, [E627K,R630G] and R630G H5 PB2 ‘627-domain’ variants were individually immobilized on a CM5 sensor chip (GE Healthcare) using an amine coupling kit until the surface density reached 700–800 response units (RU). A blank flow cell was used as a control. Wild-type and R150A NP variant in a series of concentrations were allowed to flow through the flow cells. A 2′-*O*-methylated RNA oligonucleotide with the sequence 5′ UUU GUU ACA CAC ACA CAC GCU GUG 3′ was mixed with the protein variants in some experiments to assess the effect of RNA on the NP-PB2 ‘627-domain’ interaction. SPR measurements were carried out with BIAcore 3000 at 25°C. The response in the control flow cell was subtracted from that of the experimental flow cells and the data were analyzed with BIAevaluation v. 4.1 using the model of ‘1∶1 Langmuir binding’, for the calculations of association constant, dissociation constant and the affinity of the interaction. No mass transfer effect was observed. For NP-RNA interaction study, biotinylated 2′-*O*-methylated RNA oligonucleotide with the above sequence was immobilized on an SA sensor chip (GE Healthcare) until the surface density reached 30–35 RU. NP variants in different concentrations were allowed to pass through the chip surface.

## Supporting Information

Figure S1 **Co-immunoprecipitation of wild-type and R150A mutant NP with Myc-tagged polymerase carrying either H5 or WSN(H1) PB2, in the absence of vRNA.**
(DOCX)
