# Supplementary material for: Influenza Polymerase Activity Correlates with the Strength of Interaction between Nucleoprotein and PB2 through the Host-Specific Residue K/E627
Source: PLoS One. 2012 May 3;7(5):e36415. doi: 10.1371/journal.pone.0036415 (PMC3343083; doi:10.1371/journal.pone.0036415)
Supplement: Figure S1 — Co-immunoprecipitation of wild-type and R150A mutant NP with Myc-tagged polymerase carrying either H5 or WSN(H1) PB2, in the absence of vRNA. (DOCX) [file pone.0036415.s001.docx]

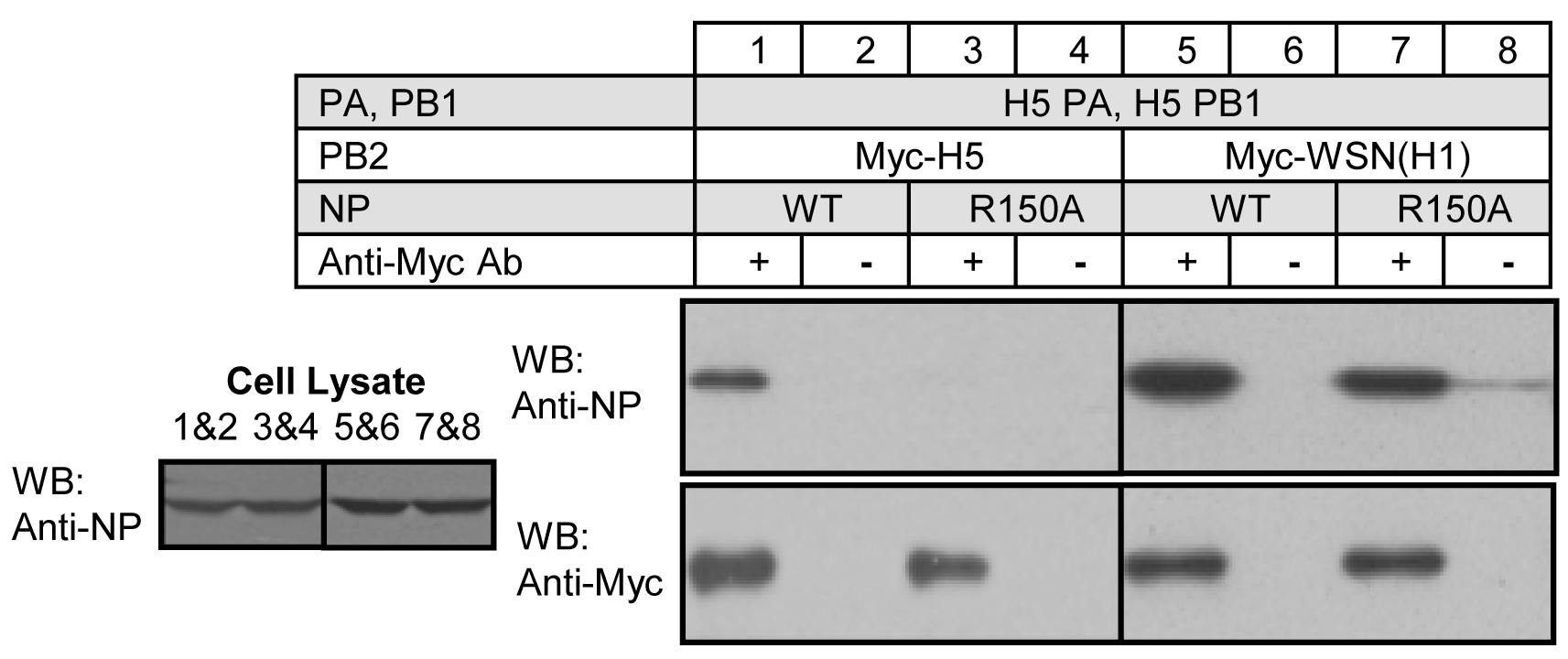


**Figure S1**

Co-immunoprecipitation of wild-type and R150A mutant NP with Myc-tagged polymerase carrying either H5 or WSN(H1) PB2, in the absence of vRNA.
